# “Nothing about us without us”: deafness in history teaching and historiography, 2015-2022

**DOI:** 10.1590/S0104-59702024000100028

**Published:** 2024-06-17

**Authors:** Valter Lenine Fernandes

**Affiliations:** i Professor, Instituto Federal de Educação, Ciência e Tecnologia Sul-rio-grandense/Universidade Federal do Rio Grande do Sul. Sapiranga – RS – Brasil valterfernandes@ifsul.edu.br

**Keywords:** Disabled people, Accessibility, History, Pessoa com deficiência, Acessibilidade, História

## Abstract

This text presents the partial results of ongoing research into deafness in history teaching and historiography between 2015 and 2022. The study problematizes the place of disabled people in top-ranking periodicals (the top two categories in Brazil) and in pedagogical projects on degree courses in history (with and without teacher-training certification) at the University of São Paulo and the State University of Campinas. These universities were chosen because they topped the ranking in a survey conducted by Folha de S.Paulo newspaper. The study observes how the Brazilian Inclusion Law (law 13.146, of July 6, 2015) is incorporated into the initial training of these professionals.

## Resumo

O texto aponta resultados parciais de uma pesquisa em andamento sobre a surdez no ensino de história e na produção historiográfica entre 2015 e 2022. O trabalho problematiza o lugar da pessoa com deficiência nos periódicos A1 e A2 e nos projetos pedagógicos de cursos de graduação em história (formação de professores e pesquisadores) da Universidade de São Paulo e da Universidade Estadual de Campinas, por conta de serem indicadas como as mais bem posicionadas no *ranking* de uma pesquisa realizada pela *Folha de S.Paulo*, levando-se em conta os critérios de articulação entre a Lei Brasileira de Inclusão, lei 13.146, de 6 de julho de 2015, e a formação inicial desses profissionais.

Este texto visa apresentar alguns resultados preliminares do projeto de pesquisa intitulado “A produção do conhecimento histórico e o ensino de história para pessoas com deficiência: ‘Nada sobre nós, sem nós’ (2015-2022)”, que tem como foco o indivíduo com surdez e suas necessidades específicas, no campo da aquisição do conhecimento histórico. O lema “Nada sobre nós, sem nós”^1^ é procedente do movimento das pessoas com deficiência da África do Sul e foi adotado em reconhecimento do fato de as próprias pessoas com deficiências promoverem diretamente os direitos humanos e o desenvolvimento de todos os sul-africanos com deficiência (citado em Sassaki, set.-out. 2007, p.21).

“Nada sobre nós, sem nós” também é utilizado no movimento pela inclusão nas palestras e nos textos do sociólogo e bioeticista inglês Tom Shakespeare – pessoa com acondroplasia e usuário de cadeiras de rodas. Em fevereiro de 2001, na conferência internacional “Deficiência com Atitude”, feita na University of Western Sydney, Austrália, ele afirmava o seguinte:

Reconhecer a perícia e a autoridade das pessoas com deficiência é muito importante. O Movimento das pessoas com deficiência se resume em falar por nós mesmos. Ele trata de como é ser uma pessoa com deficiência. Ele trata de como é ter este ou aquele tipo de deficiência. Ele trata de exigir que sejamos respeitados como os verdadeiros peritos a respeito de deficiências. Ele se resume no lema ‘Nada sobre nós, sem nós’ (citado em Sassaki, set.-out. 2007, p.20).

Nesse sentido, o objetivo geral é demonstrar a necessidade de rompimento do paradigma e da visão ultrapassada que classificava a pessoa com deficiência como incapaz, indicando aquilo de que a ciência histórica necessita para criar e consolidar espaços de conscientização sobre a inclusão. Antes da assimilação de perspectivas inclusivas nas políticas educacionais, prevalecia um modelo médico que priorizava a reabilitação para, somente depois, promover sua inserção na sociedade.

A inclusão das pessoas com deficiência na produção do conhecimento historiográfico e no ensino de história no Brasil, nos últimos sete anos, ainda tem se demonstrado incipiente, sobretudo quando falamos da acessibilidade da produção de pesquisas históricas para pessoas surdas. Antes de continuar a escrita, é necessário explicar a seguinte questão: por que usar as terminologias “pessoa com surdez”, “pessoas surdas” ou “surdos”?

Segundo Romeu Sassaki (2011), “usar ou não usar termos técnicos corretamente não é uma mera questão de semântica ou sem importância, se desejamos falar ou escrever construtivamente, numa perspectiva inclusiva”. Nesse sentido, o uso da expressão “pessoa com surdez” está atrelado ao entendimento da “pessoa com deficiência” defendido na Convenção sobre os Direitos da pessoa com deficiência, em 2006, na Organização da Nações Unidas, ou seja:

Essa Convenção diz que a deficiência é resultante da combinação entre dois fatores: os impedimentos clínicos que estão nas pessoas (que podem ser físicos, intelectuais, sensoriais etc.) e as barreiras que estão ao seu redor (na arquitetura, nos meios de transporte, na comunicação e, acima de tudo, na nossa atitude). Resumindo, entende-se que a deficiência é uma condição social que pode ser minimizada conforme formos capazes de eliminar barreiras (Mendes, 10 jul. 2020).

Já as expressões “pessoas surdas” ou “com surdez” estão relacionadas a uma diversidade de identidades surdas, sendo que, segundo Perlin (1998, p.53):

As identidades do sujeito surdo podem ser definidas como: I- Identidade Flutuante: na qual o surdo se espelha na representação da hegemonia do ouvinte, vivendo e se manifestando de acordo com o mundo ouvinte; II- Identidade Inconformada: na qual o surdo não consegue captar a representação da identidade ouvinte, hegemônica, e se sente numa identidade subalterna; III- Identidade de Transição: na qual contato dos surdos com a comunidade surda é tardio, o que faz passar da comunicação visual-oral (na maioria das vezes truncada) para a comunicação visual sinalizada – o surdo passa por um conflito cultural; IV- Identidade Híbrida: reconhecida nos surdos que nasceram ouvintes e se ensurdeceram e terão presentes as duas línguas numa dependência dos sinais e do pensamento na língua oral; V- Identidade Surda: ser surdo é estar no mundo visual e desenvolver sua experiência na Língua de Sinais. Os surdos que assumem a identidade surda são representados por discursos que os veem capazes como sujeitos culturais, uma formação de identidade que só ocorre entre espaços culturais surdos.

Para além dessas categorias definidas por Perlin (1998), cabe fazer referência à comunidade de pessoas com surdez, oralizadas e usuárias de aparelho auditivo e/ou implante coclear. Essa comunidade detém uma identidade que valoriza a tecnologia assistiva como possibilidade das ações de inclusão. Os integrantes desse grupo defendem legendas (um recurso essencial para projetos de história audiovisuais inclusivos) nos auditórios, nas salas de aula e, em reunião, a implantação de aro magnético – ao entrar no campo magnético criado pelo aro, a pessoa perceberá que o som passa a ser direcionado diretamente para o aparelho auditivo.

Apesar de existir um número significativo de pessoas com surdez espalhadas pelo território nacional, especialmente em São Paulo, maior metrópole do país, foi apenas em 2019 que o primeiro surdo oralizado e usuário de Libras, Valter Lenine Fernandes, defendeu sua tese de doutorado na área de história econômica, na Universidade de São Paulo (Saldaña, 14 ago. 2017).

Com surdez bilateral profunda, atualmente é um dos poucos docentes e pesquisadores na área de história do Instituto Federal de Educação, Ciência e Tecnologia Sul-rio-grandense e do Programa de Pós-Graduação em História da Universidade Federal do Rio Grande do Sul (Valter..., s.d.).

Dados do Instituto Brasileiro de Geografia e Estatística apontam que 5% da população brasileira é composta por pessoas que são surdas, ou seja, essa porcentagem corresponde a mais de 10 milhões de cidadãos, dos quais 2,7 milhões possuem surdez profunda (Oliveira, 23 abr. 2021).

Apesar do número expressivo de pessoas com surdez, ainda há muito a se fazer para que elas tenham acesso, de fato, ao conhecimento histórico na educação básica e nos cursos de graduação e pós-graduação. Elizabeth Tunes (2010, p.96) aponta que a ideia de deficiência pode ser entendida como um critério de preconceito: “Antes de tudo, essa noção significa falta, podendo ser parcial, transitória ou absoluta”. A partir dessa afirmação, é possível levantar uma problemática central: como as licenciaturas e os programas de pós-graduação em história vêm contribuindo para a inclusão da comunidade surda nessa área de conhecimento?

## Algumas referências teórico-metodológicas

Do levantamento de dados, foram realizados alguns recortes de periódicos da área de história classificados pelo Qualis como A1 e A2. A busca incluiu artigos ligados ao ensino de história para pessoas com deficiência ou edições acessíveis. Além disso, foram analisados dois projetos pedagógicos dos cursos da graduação em história indicados pelo jornal *Folha de S. Paulo* como referência de formação de professores e pesquisadores.

Para a análise dessas informações, as teorias das discursividades de inclusão, da manutenção da exclusão, da emergência histórica da inclusão e das atitudes sociais foram utilizadas como lente de observação, abrangendo as áreas de linguística, pedagogia e psicologia.

Por fim, estabeleceu-se um diálogo de aproximação com os estudos sobre o ensino de história desenvolvidos por Helenice Rocha. No entanto, no desenvolvimento da pesquisa, fez-se necessário atentar para o amparo das emoções pessoais de uma pessoa com deficiência e, ao mesmo tempo, para a manutenção de uma análise que vise problematizar e contribuir com o aperfeiçoamento das políticas de inclusão na área de história.

## A surdez no conhecimento histórico das revistas Qualis A1 e A2

A pouca atenção dispensada ao tema do ensino de história para pessoas com surdez e de outros segmentos da comunidade de pessoas com deficiência pode ser observada na ausência de artigos publicados por e para pessoas surdas pelas revistas científicas classificadas pelo Qualis como A1 e A2 na área de história. Isso traduz a escassa discussão sobre o lugar da surdez e da pessoa com deficiência no ensino e na produção histórica.

Inúmeros questionamentos têm sido feitos sobre o letramento na surdez. Esse tema tem sido motivo de preocupação para os pesquisadores da área de letras e tem provocado a discussão de estratégias e métodos a utilizar no processo de construção da escrita do português. Pelo fato de não ouvir, o surdo não se pauta na relação da escrita com a oralidade, daí a necessidade de afastar o grafocentrismo da escrita (Gesueli, Moura, 2006, p.111).

Isso implica a acessibilidade por meio de uma produção textual baseada em imagens ou vídeos como processo de aprendizagem, parte constituinte da publicação, o que, para a comunidade de pessoas surdas, tem um papel importante na aquisição do conhecimento histórico. Porém, o lugar ainda é de pouco mérito em periódicos ou espaços acadêmicos da história. Além disso, na maioria das vezes, os editores responsáveis estabelecem barreiras atitudinais quando um pesquisador surdo submete um artigo, pelo fato, talvez, de desconhecerem as dificuldades enfrentadas pela pessoa com surdez no processo de aquisição do português escrito.

Ademais, constata-se a não publicação em formato acessível, como determina a Lei Brasileira de Inclusão (Brasil, 7 jul. 2015), no seu art.42, inciso 1º (“É vedada a recusa de oferta de obra intelectual em formato acessível à Pessoa com Deficiência, sob qualquer argumento, inclusive sob a alegação de proteção dos direitos de propriedade intelectual”), em um grande número dessas revistas. Esse é um empecilho para o acesso à obra histórica, e, diante disso, cabem algumas perguntas: Qual o papel dos editores em relação à inclusão de pessoas com deficiência? Para quem se produz história? Há lugar para pessoas com deficiência na produção do conhecimento histórico?

Desse jeito, é necessário que as equipes editoriais garantam revistas acessíveis com o intuito de eliminar barreiras para pessoas surdas. Uma sugestão simples diz respeito aos textos das regras de publicação e/ou acesso, uma vez que a maioria das revistas não tem vídeos com intérpretes de Libras e legendas, explicando como publicar e acessar os artigos. Além da surdez, seria importante sinalizar outras necessidades específicas de pessoas com deficiência (é uma diversidade), disponibilizando um questionário no *site* para que elas pudessem indicar a necessidade das tecnologias de apoio, como proposta de eliminação de barreiras.

Assim, os pesquisadores com deficiência teriam mais condições para uma efetiva participação no acesso e na publicação de artigos. Por último, seria ideal disponibilizar um quantitativo de pessoas com deficiência nas equipes editoriais. Maura Lopes e Eli Fabris (2013, s.p.) verificam esse processo da falta de clareza da temática da inclusão como uma lógica moderna e kantiana de que “o sujeito capaz de conduzir a si próprio é aquele que já atingiu a maioridade ou foi conduzido pela boa educação e disciplina durante a menoridade”. Dessa maneira, é emergente a articulação de estratégias para que o lugar da pessoa com deficiência seja garantido e se evite a reprodução de um pensamento que aproxima “a criança, o jovem, o deficiente, o louco, entre outras figuras que aprendemos a definir, em diferentes tempos e espaços, como incapazes de racionalidade e autocondução” (Lopes, Fabris, 2013, s.p.).

Nesse caso, é necessário situar algumas temporalidades históricas da pessoa com necessidades específicas no Brasil, para verificarmos a perpetuação da inclusão ou da exclusão na produção historiográfica e no ensino de história. Segundo Gilberta Jannuzzi (2010, s.p.),

é possível apontar desde o Brasil colônia, tanto a lei excludente de direitos dos cidadãos ‘diferentes’ como também alguma possibilidade de participação social, na medida em que, recolhidos com os abandonados em asilos, alguns receberam instruções que os habilitaram a viver em um meio social mais amplo. Posteriormente, já em instituição especializada, portanto, diferenciada, trazendo a marca da segregação, havia também a possibilidade de vislumbrar as grandezas do saber, por meio de métodos e técnicas especializadas, como foi proposto em dois institutos no século XIX: o dos cegos e o dos surdos, atualmente conhecidos como Instituto Benjamin Constant (IBC) e o Instituto Nacional de Educação dos Surdos (Ines), bem como algumas escolas que surgiram para atender os ditos ‘deficientes mentais’ (destaques no original).

Na atual realidade do Brasil, o direito à educação de pessoas surdas está garantido na Lei Brasileira de Inclusão, número 13.146, de 6 de julho de 2015; no decreto federal 5.626, de 22 de dezembro de 2005; na lei 10.098, de 19 de dezembro de 2000; e pela lei 10.436, de 24 de abril de 2002, que reconhecem os surdos como um grupo minoritário e algumas identidades surdas com uma língua própria, que é a Libras, a Língua Brasileira de Sinais.

Nos principais centros de produção historiográfica, observamos uma relativa ausência de políticas e/ou pautas que discutam a inclusão de um conhecimento histórico acessível para pessoas surdas. Esse aspecto impacta no processo de elaboração de materiais que possam contribuir para um ensino de história crítico e acessível para pessoas com deficiência, da educação básica ao ensino superior.

## A pessoa com deficiência no projeto pedagógico de curso

Dos artigos 27 ao 30, a Lei Brasileira de Inclusão assegura à pessoa com deficiência um sistema educacional inclusivo em todos os níveis de aprendizado ao longo de toda a vida, de forma a alcançar o máximo desenvolvimento possível de seus talentos e habilidades físicas, sensoriais, intelectuais e sociais, segundo suas características, interesses e necessidades de aprendizagem. Destacamos aqui alguns incisos do art. 28 da referida lei: III, IV, V, VI, VII, XI, XII, XIII e XIV (Brasil, 7 jul. 2015).

No que diz respeito ao ensino de história, como espaço de produção do conhecimento, Erinaldo Cavalcanti e Helenice Rocha (2020, p.5) definem, na apresentação do dossiê “O ensino de história entre lutas, alegrias e esperanças”, que,

talvez, possamos falar que existe algum consenso que a História – como lugar de produção de saber e espaço de formação docente –, precisa ressignificar as matrizes curriculares dos cursos de licenciatura ... Ou seja, a formação docente, em História, precisa ocupar os proscênios do centro de interesse dessa ciência. As questões que envolvem as diferentes narrativas que disputam a produção de sentido, no cotidiano de homens, mulheres, crianças e adolescentes (o potencial público a ser atendido pelo professor de História) precisam ser objeto de formação durante a formação inicial desse professor.

Ao verificar alguns projetos pedagógicos dos cursos da Universidade de São Paulo (USP) e da Universidade de Campinas (Unicamp), percebeu-se que não se encontra uma formação inicial do licenciado ou bacharel em história que discuta temas emergentes sobre a pessoa com deficiência, ainda que no capítulo IV da Lei Brasileira de Inclusão haja a previsão, em todas as modalidades de ensino, da obrigatoriedade de uma educação acessível. Sendo assim, nessa fase da pesquisa, as grades de organização acadêmicas dessas instituições foram selecionadas para efeito das primeiras discussões a respeito da aplicação prática da legislação. As demais universidades brasileiras não serão contempladas em um primeiro momento, pois o intuito é gerar problemáticas para que outras pesquisas sejam realizadas acerca da temática. Cabe lembrar que, em 2019, as supracitadas universidades ocupavam o *ranking* das duas melhores graduações em história, conforme a pesquisa realizada pelo jornal *Folha de S.Paulo* (Ranking..., 2019).

O projeto pedagógico do curso de graduação em história da Unicamp oferece duas graduações em história (licenciatura e bacharelado) e duas ênfases (ênfase em história da arte e ênfase em patrimônio histórico e cultural). São dois campos de conhecimento que devem contemplar a obrigatoriedade da acessibilidade. Porém, o projeto pedagógico menciona de forma genérica o conceito de inclusão, negligenciando a expressão “pessoa com deficiência” e a citação da Lei Brasileira de Inclusão. Não existe uma disciplina que discuta o ensino de história e a acessibilidade das pessoas com necessidades específicas (Projeto..., 2018).

Greciely Cristina da Costa (2014, p.134) sugere que a inclusão seja abordada de forma abrangente, mas com o devido cuidado de contextualização, para que “não se estabeleça uma relação condicional, fazendo com que o sujeito, afetado pela ilusão da inclusão, no processo de individuação, se identifique como excluído, buscando, almejando e se responsabilizando pela sua própria inclusão”. É necessário questionar até que ponto o uso de discursos coletivos que abordam a construção do conhecimento histórico e o ensino de história para pessoas com deficiência são uma tentativa de negação da inclusão e de transferência de responsabilidade da ausência de pautas para a pessoa com necessidades específicas.

O Projeto Acadêmico 2019-2023 da graduação do Departamento de História da USP, segundo documento publicado no *site* da Faculdade de Filosofia, Letras e Ciências Humanas, tem como princípios norteadores:

Indissociabilidade entre ensino e pesquisa: alunos e docentes da comunidade acadêmica do Departamento de História transitam ininterruptamente entre um e outra em aulas, laboratórios, estágios, seminários, pesquisas de campo e em arquivos, operando críticas historiográficas, discussões teórico-metodológicas, produção de textos que articulam uma formação voltada para o pesquisador em História e para o (futuro) professor, tudo a um só tempo (Projeto..., s.d., p.3).

...

É relevante também na formação, igualmente constante, de pesquisadores para museus, arquivos, bibliotecas, e centros de cultura e de patrimônio históricos públicos (municipais, estaduais e federais) e privados (p.1).

No projeto acadêmico do Departamento de História dessa universidade, existe uma ausência de indicativos para a inclusão e acessibilidade de pessoas com deficiência para a formação do pesquisador e do professor de história. Em linhas gerais, a ideia aqui não é desmerecer a formação da USP, mas problematizar os lugares da pessoa com deficiência no planejamento de formação dos futuros pesquisadores e docentes, que terão como desafio a emergente aplicação desse conhecimento por meio do Marco Legal, de 2015, e por conta de atitudes sociais inclusivas.

Indinéia Paixão constatou em sua dissertação, defendida no Mestrado Profissional em História da Universidade do Estado da Bahia, que são poucos os estudos sobre o ensino de história para surdos. A historiadora apresentou um quadro com as informações mais recentes sobre essa produção e percebeu que, de 2018 até 2021, são aproximadamente 19 trabalhos, a maioria distribuída em anais de eventos e em três dissertações de mestrado. Assim, ela afirma que

podemos constatar que já existe um caminho sendo trilhado, no sentido de colocar em pauta as discussões e práticas que viabilizem o Ensino de História para estudantes surdos. No entanto, ainda temos um longo caminho pela frente, sobretudo no que diz respeito a essas discussões chegarem ao chão da escola e estimularem a criação de novas estratégias pedagógicas para o Ensino de História com discentes surdos(as) (Paixão, 2022, p.54).

Diante dessa constatação por meio dos estudos de Indinéia, verificou-se a necessidade de aprofundar a análise dos projetos pedagógicos dos cursos das graduações em história, além da sua relação com a ausência de articulação da formação inicial do pesquisador e do professor, para pensar estratégias no ensino de história para pessoas com deficiência e, especificamente nessa primeira fase da pesquisa, para pessoas com surdez. Outro fato importante é que isso gera uma realidade de esquecimento e/ou de ausência de consciência de novas pesquisas que destacam a relevância do lugar ou de práticas inclusivas da pessoa com deficiência nas revistas de história classificadas pelo Qualis como A1 e A2.

## Os próximos sentidos

As fases posteriores da pesquisa têm como objetivo ampliar um estudo sistemático para o quantitativo de revistas A1 e A2 da área de história, que possuem uma prática editorial inclusiva, argumentando sobre a importância do lugar da pessoa com deficiência na produção histórica. Outro ponto de investigação será verificar o quanto essas equipes editoriais estabelecem discussões em relação à Lei Brasileira de Inclusão, número 13.146, de 6 de julho de 2015. Nesse caso, a problemática norteadora é saber se é necessário conhecer a lei para o aumento da adesão.

Pretende-se, assim, mapear práticas e políticas adotadas pelas revistas para o acesso de pessoas com necessidades específicas. É preciso entender, também, por que não é instituída nos projetos pedagógicos de cursos ou projetos acadêmicos uma discussão mais aprofundada sobre o ensino de história e as pessoas com deficiência, na formação inicial de professores e pesquisadores, podendo afirmar que esse é um desafio para as pessoas com deficiência.

Ainda no que diz respeito à inclusão e ao direito de superação de barreiras de acesso ao patrimônio histórico, obras intelectuais acessíveis estão previstas em lei; portanto, a diminuição dos obstáculos atitudinais deveria ser instituída nos textos de organização das matrizes curriculares.

Em outra etapa da pesquisa, será observado o lugar da Associação Nacional de História no exercício de espaços permanentes de debates para a garantia de direitos da pessoa com deficiência na produção historiográfica e no ensino de história.

## NOTAS

1 O Grupo de Usuários de Estratégias, do Ministério da Saúde da Grã-Bretanha, é formado por pessoas com dificuldades de aprendizagem, participantes das organizações People First, Mencap, Change e Speaking Up. O Grupo adotou o lema “Nada sobre nós, sem nós” para exigir a inclusão de pessoas com dificuldade de aprendizagem em todos os serviços públicos. O grupo de estratégias era constituído com representantes de todo o país e era uma espécie de órgão consultivo que, juntamente com o Ministério da Saúde, elaborava uma estratégia para pessoas com dificuldades de aprendizagem (Sassaki, set.-out. 2007).
